# Validation of a Spectral Flow Cytometry Single-Tube Panel for the Clinical Diagnosis and Follow-Up of Children and Adolescents with B-Cell Acute Lymphoblastic Leukemia

**DOI:** 10.3390/cells13221891

**Published:** 2024-11-15

**Authors:** Gonzalo García-Aguilera, Ana Castillo-Robleda, Alejandro Sanz, Manuel Ramírez

**Affiliations:** 1 Advanced Therapy Research Unit, Hospital Universitario Niño Jesús, 28009 Madrid, Spain; gonzalo.garcia.externo@salud.madrid.org; 2 Clinical Diagnosis Unit, Hospital Universitario Niño Jesús, 28009 Madrid, Spain; acastillor@salud.madrid.org (A.C.-R.); alejandro.sanz@salud.madrid.org (A.S.); 3 Pediatric Hematology & Oncology, Hospital Universitario Niño Jesús, 28041 Madrid, Spain

**Keywords:** spectral flow cytometry, B-ALL, clinical diagnosis

## Abstract

The ability of flow cytometry to identify and quantify the presence of cell populations defined by their expression profile of specific markers has made this technique a powerful and routinary tool in clinical diagnostic practice. Specifically in the field of hematological malignancies, flow cytometry allows the identification of the correct type and lineage of each patient’s disease and also sensitively quantifies the presence of the disease at precise moments during treatment, that is, levels of measurable residual disease (MRD). The quantification of MRD by flow cytometry has allowed the adaptation of tailored therapies to patients, contributing to the improvement of the results of the different protocols in recent decades. In this context, our objective in the present work was to evaluate the potential impact that spectral flow cytometry can provide compared to conventional cytometry, which is the one usually used in clinics. We present here a comparative study of both technologies, spectral versus conventional flow cytometry, in primary samples corresponding to the diagnosis and follow-up of children and adolescents with acute lymphoblastic leukemia. Our initial experience demonstrates the feasibility of incorporating spectral flow cytometry into the routine workflow of a reference laboratory.

## 1. Introduction

One of the main and most widespread practical applications of flow cytometry is the detection of different cell populations. Blood samples and other derivatives (bone marrow, apheresis products) can be easily processed for accurate and reliable detection of the leukocyte or lymphocyte subpopulations they contain, as well as for exhaustive phenotypic characterization of certain aberrant populations. These studies are of great clinical importance (diagnosis and monitoring of malignant hematological diseases, detection of immunodeficiencies, evaluation of response to immunotherapy treatments, development of cell-processing platforms in the context of hematopoietic progenitor transplantation) [1].

Conventional flow cytometry has been used for decades for this purpose, progressively expanding knowledge of the different immune cell subpopulations and the antigens expressed in the various cell populations and which therefore allow them to be detected, quantified, and monitored. Technological development has also facilitated the implementation and continuous review of study protocols using flow cytometry, which has reached a turning point with the arrival of spectral flow cytometry [2].

Spectral flow cytometry provides the ability to detect a much larger number of cell markers per single test tube than conventional cytometry, allowing an image of the complete emission of light from each fluorochrome instead of being limited to the maximum emission peak. The main advantages of this greater number of detectors are the possibility of combining a greater number of fluorescences than has usually been employed in clinical laboratories, as well as detecting with greater precision and reliability the populations that express each marker analyzed.

Spectral flow cytometry has been working in experimental laboratories for research purposes [3] for quite a long time. However, the clinical diagnosis environment is very different from that of the research laboratory in many aspects [4]: response times, workflows, pre-analytical phase, obligation to adhere to standardized work protocols and use of reagents, among others, which usually increases complexity (at least initially) and cost. There is a need to carry out validation studies before transferring spectral flow from the bench to clinical practice in order to incorporate it into the routine day-to-day life of a diagnostic laboratory [5,6]. We present here a comparative study of both technologies, spectral versus conventional flow cytometry, in primary samples corresponding to the diagnosis and follow-up of children and adolescents with acute lymphoblastic leukemia.

## 2. Materials and Methods

### 2.1. Flow Cytometers

We used FACS Canto II (Becton Dickinson) (Madrid, Spain) and Northern Lights (Cytek) (Madrid, Spain) flow cytometers for this comparative study. Preparation, quality control, maintenance, and cleaning of each apparatus were performed daily, adhering to the manufacturers’ recommendations and reagents for quality control. Internal standard operating procedures were followed along this study, under ISO15189 accreditation (obtained in 2024).

### 2.2. Fluorochromes and Panel Design

Leukocyte markers and the corresponding antibodies and fluorochromes were selected based on their contribution in separating B-cell progenitor acute lymphoblastic leukemia (BCP-ALL) cells from normal/regenerating BCP cells (Supplementary Table S1). For conventional cytometry, 8-color antibody tubes were selected, containing antibodies (CD19, CD45, CD34, CD10, and CD20) selected upfront as backbone markers because they allow for the appropriate characterization of BCP subpopulation maturation. These markers were combined with additional ones that detect antigens known to be aberrantly expressed by many BCP-ALLs, i.e., CD9, CD123, CD66c, CD73, and CD304, among others. These markers appear to be most promising based on the level and frequency of overexpression and their stability during therapy.

We designed a 24-color single-tube panel for spectral flow cytometer that integrated all the markers defined for conventional flow. Choosing antibodies and fluorochromes was based on the varying levels of brightness and antigen densities. A virtual simulation of the complexity and similarity between the different antibodies was performed (avoid designing panels with antibodies that are excessively similar to each other), using the Cytek Cloud (https://cloud.cytekbio.com/), adding all the desired fluorescences, and analyzing how they behave when combined. In order to characterize B-ALL in its entirety, we had to use fluorochromes that are close in the emission spectrum. We verified that there were no two fluorescences at the same maximum emission peak and that the complexity index was clearly lower than the number of fluorochromes used and no antibody combination had a similarity greater than 0.95. Fluorochromes were selected based on the density, intensity, and rarity of the marker.

The antibodies used for flow cytometry in this validation study were as follows:

### 2.3. Bone Marrow Samples

The samples for the present project were marrow aspirates collected from May 2013 to December 2022 at the Hospital Infantil Universitario Niño Jesús. All patients signed the corresponding informed consent to donate the study samples (clinical study internal code R-0017/13, approved on 8 May 2013). Samples were either stained on the same day the bone marrow was received, or frozen after Ficoll separation and stored in liquid nitrogen for further use.

### 2.4. Staining Protocol

We followed an erythrocyte bulk-lysis protocol that allowed the acquisition of high cell numbers. The final volume contained 100 µL of sample and the mixture of antibodies. During optimization, some antibodies were sequentially added to the cocktail, as explained below. Antibodies were centrifugated at maximum speed before use to eliminate aggregates. Tubes were incubated for 20 min at room temperature in the dark and washed with 3 mL of PBS. Samples were resuspended in PBS with 1% FBS and 2 mM EDTA and acquired in the respective flow cytometers.

### 2.5. Gating and Data Analysis

Flow cytometry standard (FCS) files from both cytometers were manually gated to remove doublets, debris, and dead cells, using Diva (BD Biosciences software Version 10.8.1) (Madrid, Spain) or Spectroflow (CLC Version 1.0.3, Cytek) (Madrid, Spain). Bone marrow populations, including myeloid, monocytic, and lymphoid subsets, were identified on the basis of CD45 and side-scatter combination (Supplementary Figure S1). Studies done on samples at diagnosis easily identified the population containing leukemic cells on the CD45 and side-scatter dot plot. The presence of aberrant marker expression, maturational asynchrony, discordant intensity of expression, or expression on non-hematopoietic markers (i.e., the NG2 antigen) was individually identified for each patient so that a leukemia-associated immunophenotype (LAIP) could be defined based on the expression of specific markers. This LAIP was used for the evaluation of residual leukemic cells in the follow-up studies. In all cases, possible leukemic cells were ultimately back-gated on the forward-scatter (FSC)/side-scatter (SSC) and CD45-SSC plot to check that the events formed a uniform cluster.

## 3. Results

### 3.1. Antibody Titration

**Figure 1:**
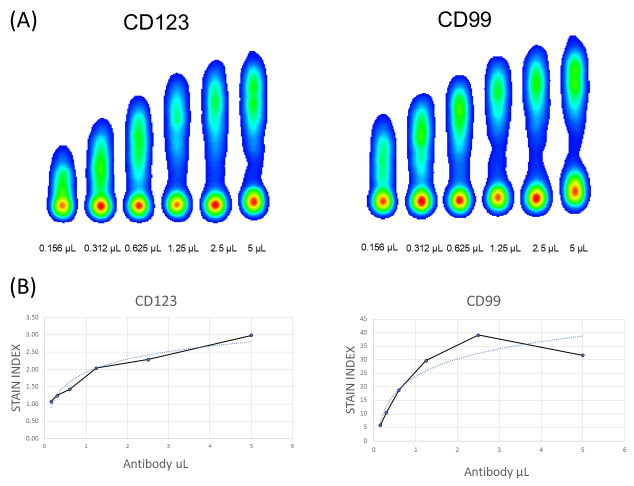
Example of antibody titration. (**A**) Density plots showing the titration of CD123 and CD99 with different amounts of antibody. (**B**) Curves of the stain index of the antibodies. Graphs showed the µL required to saturate each marker.

We first titrated the antibodies chosen for the flow cytometry B-cell ALL panel, following Cytek Antibody Titration Protocol to determine the optimal volume of each antibody. We used frozen diagnostic samples of pediatric B-ALL (Figure 1A) for this purpose. Thawed cells were resuspended in RPMI for 2 h to recover the superficial markers. Unstained plus single-stained tubes (SST) with decreasing amount of each antibody (5 × 10^−1^ dilutions, 6-point titration for each antibody, Figure 1A) were run independently and the stain index (SI) was calculated with the formula SI = $\frac{MFIpos-MFIneg}{2\times rSDneg}$ (Figure 1B), where *MFIpos* is the median fluorescent intensity of the positive population, *MFIneg* is the median fluorescent intensity of the negative population, and *rSDneg* is the robust standard deviation of the negative population. The optimal volume was chosen as the lowest that achieved the highest SI. A representative example of the stain index calculation is shown in Figure 1. Final volumes for each antibody in the panel are shown in Table 1.

### 3.2. Single Versus Multiple Stain

**Figure 2:**
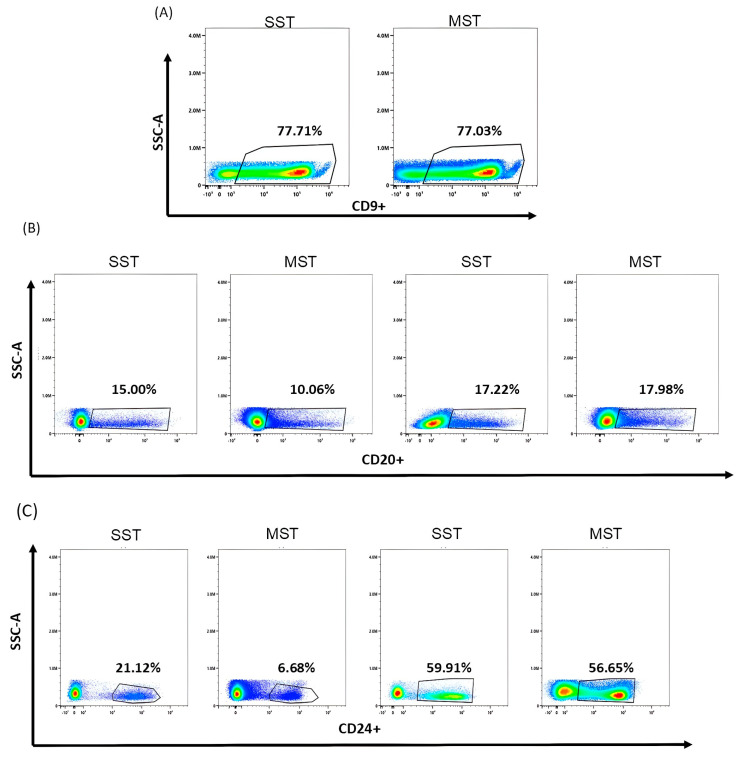
Single versus multiple stain. (**A**) Example of CD9 staining in SST and MST. (**B**) Example of CD20 staining; the left 2 plots show significant loss of detection of the positive population in the sample when comparing SST versus MST; plots on the right showed how increasing antibody volume corrected the error. (**C**) Example of CD24; the left 2 plots show discordant results between SST and MST, the right plots show results after sequential staining.

Once each antibody had been titrated, we prepared SSTs with the chosen volume plus an unstained tube and a multiple-stain tube (MST). We ran each SST tube and activated the spectral unmixing algorithm for deconvolution, before acquiring the MST. We next compared each individual SST with the MST to check that the same percentage of positive events were detected when using 24 markers in the same sample (Figure 2).

We calculated the repeatability (accuracy) of the method by comparing the mean fluorescence intensity (MFI) of each marker obtained in three separate experiments using cells (Table 2). We did not find significant differences between technologies. This comparison was not done when using beads for the setting up.

This comparison allowed us to fine-tune the staining protocol. We found that most of the previously chosen volumes worked at similar levels in the SST and the MST (Figure 2A). However, for markers such as CD20, MST resulted in a significant loss of the positive population detected in the SST. The problem was solved by increasing the antibody volume to the level immediately above the SI (Figure 2B). In other markers, we found a lower proportion of positive events in the MST compared to the corresponding SST even at the highest recommended volumes (5 μL). Suspecting steric problems for these markers, we conducted a sequential staining. We first labelled the sample with these antibodies (CD52, CD304, CD73, CD24, and CD20), incubated at room temperature in the dark for 10 min, and then added the remaining antibody mixture. This sequential staining strategy resulted in comparable proportions of positive events in the SST and MST (Figure 2C).

We next evaluated dot plots combining two fluorescences to check for spreading and the need of optimizing the compensation matrix generated by the unmixing algorithm. Minor unmixing errors were corrected modifying the spillover matrix (i.e., CD9-cF V450 and CD52-cF V420). Compensation was reviewed after the acquisition of the samples. Finally, we found a major spreading problem related to the CD66c cFV780 antibody initially used in our panel, not resolvable by the fine-tuning strategies previously described (Supplementary Figure S2).

**Figure 3:**
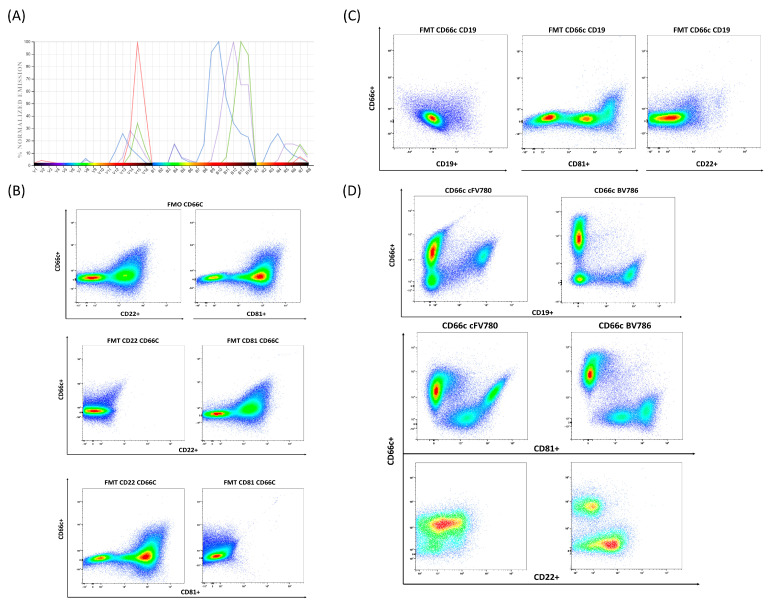
Resolving spreading errors. (**A**) Spectrum emission of CD66c-cFV780 (Red), CD22-cF BYG710 (blue), CD19-cF BYG781 (green), and CD81- cF BYG750 (purple). The X-axis shows the emission channel. (**B**) FMO of CD66c. The CD66c detector was plotted against CD22 and CD81. FMT of CD66c and CD22. FMT of CD66c and CD81. (**C**) FMT of CD66c and CD19. (**D**) Comparison between CD66c cFV780 and CD66c BV786, plots showing how the unmixing error was corrected with the new fluorochrome conjugation.

We checked the spectrum emission to rule out the fluorophores that might have the highest similarity with the cFV780 and could generate the spread detected (Figure 3), and identified cF BYG710 (conjugated to CD22), cF BYG781 (conjugated to CD19), and cF BYG750 (conjugated to CD81) as the major candidates. We next ran a fluorescent minus one (FMO) tube (MST tube without adding the CD66c cFV780 antibody) but the spread persisted. We next tested various fluorescent minus two (FMT) MST tubes in which paired combinations of the four mentioned antibodies were absent. Using this strategy, we eventually identified CD19 cF BYG781 as the cause of the spreading problem. Given the crucial role of CD19 in B-cell ALL and the fact the CD66c is only expressed in a proportion of B-cell ALL cases, we decided to test a new fluorescent tag for CD66c, BV786. We observed that with the new BV786 label, the spread disappeared when we plotted CD66c vs. CD19 (Figure 3). We also checked for spread on CD22 and CD81 with the new fluorophore for CD66c and found no major spread in both plots.

### 3.3. Validation of the B-ALL Panel with Primary Samples at Diagnosis

Once the panel was finally optimized, we validated it by running primary B-ALL samples from diagnosis/relapses, in parallel in a conventional flow cytometer and in a spectral one. All samples were processed and acquired by the same technician personnel on the same day, and analyzed independently by two faculties. Panel performances were compared by evaluating the population frequencies and visual staining patterns on each instrument. Twenty-seven fresh B-ALL samples were studied, the intensity of each marker was catalogued as negative, dim, positive, or highly positive. A leukemia-associated immunophenotype (LAIP) was defined based on the expression of specific markers. Although the two systems utilize different laser powers, types of detectors, and methods of signal capture, we sought to determine if the population frequencies and visual staining patterns produced by each instrument were comparable. The main immune cell populations generated by the two panels were gated for each bone marrow sample on both instruments and calculated as frequencies of CD45+ leukocytes. Expression levels for each marker were catalogued as negative, dimly positive, positive, or highly positive. The threshold is typically set based on the negative population for each marker shown in a plot. No major differences were seen when comparing each marker. Minor differences in intensity expression were found in several markers when comparing both technologies, as shown in Supplementary Table S2. Discrepancies varied depending on the markers and the samples. We finally conducted 517 paired comparisons, finding 46 discrepancies (8.9%) between conventional and spectral flow cytometry when cataloguing intensity expression. We found no differences in expression intensities in six markers (CD2, CD9, CD19, CD38, CD81, and NG2), seven markers for which spectral flow cytometry gave higher intensities compared to conventional flow cytometry (CD15, CD10, CD20, CD24, CD45, CD58, and CD99), five markers for which conventional flow cytometry gave higher intensities compared to spectral flow cytometry (CD13, CD33, CD66c, CD123, and HLA-DR), and three markers for which discrepancies in intensities were evenly distributed between both technologies (CD22, CD34, and CD52).

We compared the expression level of each marker and calculated the ratio of overestimation (intensity in spectral greater than in conventional) and the ratio of underestimation (intensity in spectral greater than in conventional) found in our study (Table 3). Despite the differences found at the level of individual markers, we did not find discrepancies between technologies in defining the LAIP for each leukemic case. The reproducibility of leukemic population frequency output between the Cytek Northern Lights and BD FACSCanto II instruments gave us confidence that the development of the panel was flexible enough to accurately phenotype BCP-ALL cells for clinical purposes.

### 3.4. MRD Simulation

**Figure 4:**
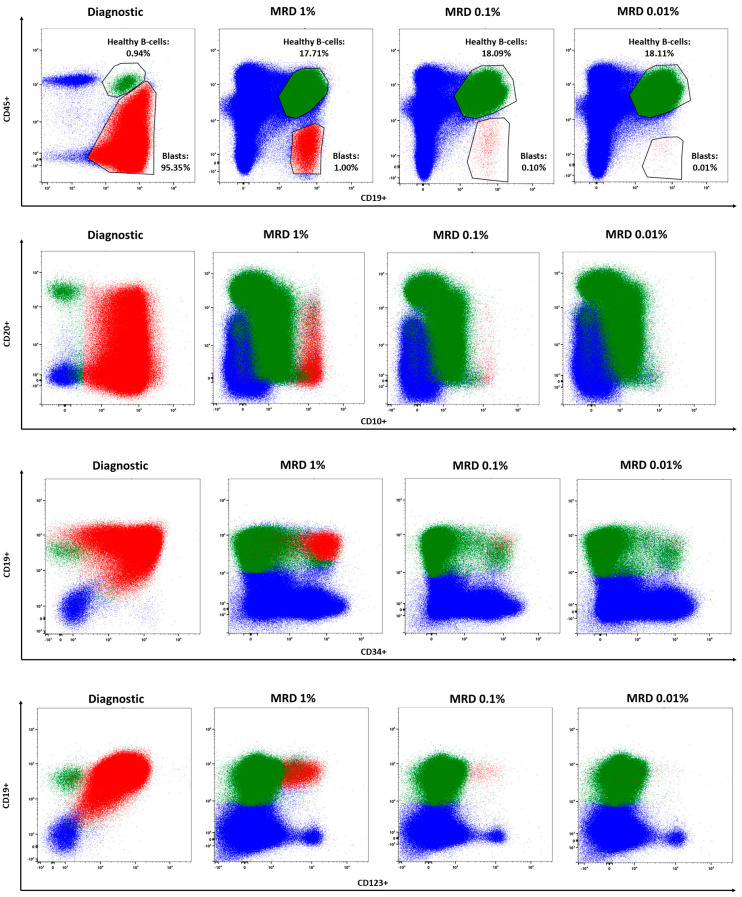
Quantitation of measurable residual disease (MRD). Detection of leukemic cells in different concentrations diluted in a healthy bone marrow. Myeloid and T-cells (blue), healthy B-cells (green), and blasts (red).

We also evaluated the panel’s ability to detect and quantify the levels of measurable residual disease (MRD). We first “simulated” MRD by serial dilutions of B-ALL cells from a diagnostic sample in healthy peripheral blood cells, down to 0.01%. We acquired 2 million total events and searched for the LAIP to obtain a quantitative estimation of MRD levels (Figure 4).

Using this strategy, we detected leukemic residual cells down to a 10^−4^ concentration. We next validated the panel for MRD quantitation by running B-ALL samples during therapy in parallel in a conventional and a spectral flow cytometer.

**Figure 5:**
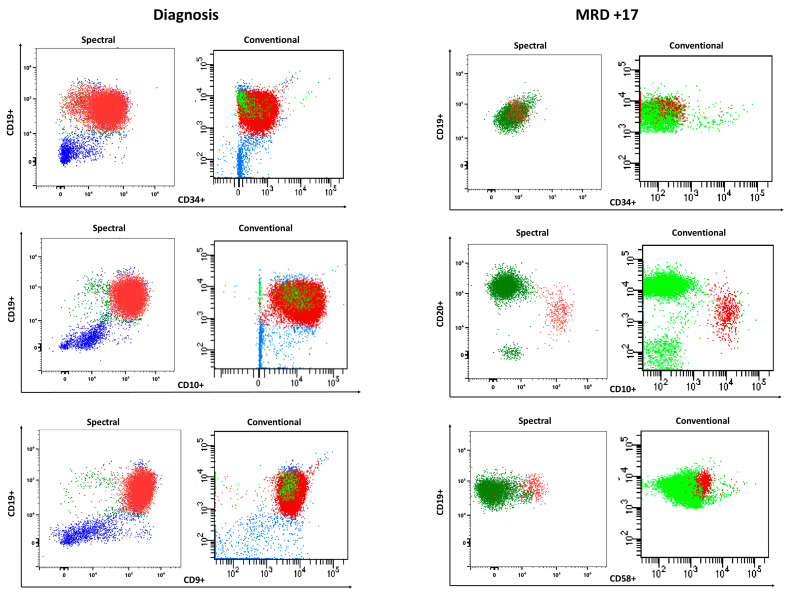
MRD quantitation in clinical samples. Comparison of diagnosis and day +17 studies of marrow aspirates from a patient using conventional and spectral flow cytometry. Red dots correspond to leukemic blasts, green dots correspond to healthy B cells. Blue dots correspond to Myloid and T cells.

During 6 months, BM samples obtained during follow-up of BCP-ALL patients were processed in pairs and analyzed on a dual platform, with the eight-color panel on a conventional cytometer and with the twenty-four-color panel on a spectral cytometer (Figure 5).

**Figure 6:**
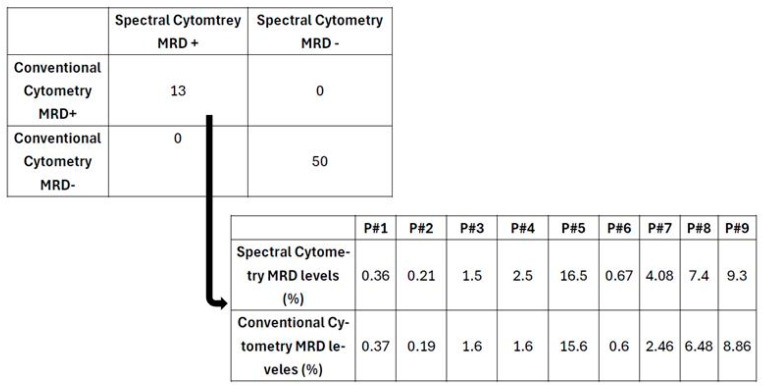
Paired comparison of MRD determinations. Above, total results. Below, levels of discrepancies in 9 studies of MRD quantitation of patients with BCP-ALL.

The selected BCP-ALL MRD tubes were prepared using an optimized bulk-lysis protocol. We ran 69 paired analyses and found that MRD results were concordant, both in cases of MRD considered positive and in cases of undetectable MRD (Figure 6).

Nine out of sixty-three studies (14%) resulted in a minimum difference of less than one logarithm, all in positive results by both techniques. These nine cases initially scored positive by both FCM-MRD conventional and spectral but they were reanalyzed. The minimal differences were related to the number of events acquired, to the cleaning of debris, and the elimination of doublets (Supplementary Figure S3).

## 4. Discussion

The arrival of new technology and new developments that significantly improve the state of the art in laboratories dedicated to clinical diagnosis is a process that requires a validation period, necessary to reliably verify the performance of the new capabilities [2,4,5,6]. The innovations must demonstrate significant advantages for the analyst, while ensuring the same degree of sensitivity and accuracy as conventional techniques when obtaining the result. The clinical environment is very different from that of the research laboratory in many aspects: response times, workflows, pre-analytical phase, obligation to adhere to standardized work protocols, and use of reagents, among others, which usually increases complexity (at least initially) and cost. It is not unusual for the first experiences with new technologies to occur in research laboratories long before being transferred to the environment of a clinical diagnostic laboratory.

Our laboratory has initiated the project of evaluating spectral flow cytometry in the clinical context, as a previous and necessary step before its incorporation into the diagnostic routine. This initial phase that we discuss in this work has been carried out first in a research laboratory and then adapted to the workflow of a clinical laboratory, all within the same academic institution. The development of the panel for the diagnosis and monitoring of children and adolescents with B-ALL using spectral flow cytometry is a process not particularly complicated in practice, since the principles applied do not differ greatly from those used for conventional cytometry [7]. Antibody titration, adjustments of the deviations in the multi-label tube, and the need for compensation of fluorescence are all phases that are carried out in either of the two technologies. The inclusion of a high number of antibodies in the same tube can cause the masking of some epitope due to steric impediments, which we have resolved by means of sequential labeling. There may also be a spread that cannot be corrected during unmixing, when there are several fluorophores with very similar emission spectra, a case presented in this work. In these situations, it is essential to compare the FMO and FMT options to identify the causes of the dispersion problem and be able to correct it. Discrepancies in intensity expression for the different markers were eventually minimal once we finely adjusted all the markers in the panel.

In our case, most of these optimizing steps were performed with primary cells stored in the research laboratory collection, which is advisable but not always within the reach of clinical laboratories. It is well-known that the freezing process can alter not only the number of viable cells but also the expression of certain surface markers (due to the normal recycling of proteins, at least). To limit the impact of this effect, we always let the cells recover at room temperature for at least 2 h after thawing. Under these conditions, cells maintain their viability during the processes of titration and acquisition. The alternative of beads, which we use in very few situations, has an adequate performance for this process of setting up the test conditions.

The application of the designed and optimized panel in the diagnosis of patient samples offered results completely concordant with those obtained with our usual four-tube method. It should be noted that the assignment of the type and lineage of the leukemia, the screening tube, was always performed as a previous step in a conventional cytometer. Markers used for BCP-ALL characterization such as IgM or TdT were included in the screening tube. It is also important to underscore that the panel described here was generated for bone marrow samples, but it is applicable to peripheral blood cell samples, as we have done in the follow-up of some cases.

The minimal differences found in the intensity of expression of some markers between the two types of cytometry never resulted in the assignment to different stages of differentiation, that is, pro-B, pre-B, B-common. More importantly, the immunophenotype associated with leukemia was identified in each case with the same markers, regardless of the technology used. The study of primary samples allowed us to validate the application of this spectral flow cytometry panel in clinical practice in our laboratory.

The ability of spectral flow cytometry to include a large number of markers in a single tube makes it very attractive for clinical laboratories dedicated to quantifying the levels of measurable residual disease in patients with hematologic malignancies who are receiving treatment [1,8]. It is at these times, when chemotherapy-induced cell death and the regeneration of healthy hematopoiesis are occurring in the bone marrow, when the accurate quantification of viable malignant cells present among the vast majority of healthy cells becomes of capital importance, given that individualized clinical decisions will be made regarding the treatment of each patient. The quality of the sample to be studied and the absolute number of cells to be processed profoundly affect the performance of cytometry, but they are variables that cannot be controlled by the laboratory. Circumstances such as the patient’s condition at the time of the bone marrow aspiration, the volume of the sample, the degree of contamination with peripheral blood, or the conditions and duration of the sample shipment, are always prior to the cytometry study and can have a negative effect on the level of sensitivity finally achieved [2]. In this scenario, most laboratories today divide these samples into (at least) two aliquots to assemble two cytometry tubes, with antibodies serving as backbone in both tubes to which additional antibodies are added, different in each tube, directed to markers that distinguish malignant cells from healthy progenitors [9]. The need for these two tubes not only makes it difficult to achieve a sufficiently high number of events in regenerating marrows to ensure a sensitivity of 10^−5^, for example [9,10], but also imposes limitations on the analysis. The existence of two tubes forces an estimate to be made since not all the markers are found in the same sample. Spectral flow cytometry promises to solve these limitations of standard practice since all the material obtained can be analyzed in a single tube containing all the antibodies with which the diagnosis was made and the LAIP population was defined. Our validation work has allowed us to confirm that spectral flow cytometry reached the levels of sensitivity required for MRD quantification and performed at the same level as conventional flow. The comparison in MRD quantitation gave a very high level of concordance between both technologies; minor differences could be explained by the difference in total viable leukocytes when excluding debris and doublets performed in the two analyses’ platforms. The spectral cytometer has two detectors for SSC, with the SSC-B-A/SSC-A dot plot allowing better discarding of erythroid cells (Supplementary Figure S3). In any case, differences in MRD levels were always minimal; in no case did the discordance separate results at the level of the cut-offs described in the treatment protocols for score risk. The impact of applying spectral flow cytometry in the quantitation of MRD levels at the end of induction and the end of consolidation, when cutoffs are set at 10^−3^ and 10^−4^ in most therapy protocols [9,10], may be clinically important, given the higher sensitivity of spectral flow cytometry for dim signals plus the capacity to include all events in a single tube.

In summary, we present here experimental observations of our initial experience in validating spectral flow cytometry in the clinical settings of a reference laboratory dedicated to the diagnosis and follow-up of children and adolescents with acute lymphoblastic leukemia. When moving into the context of clinical diagnostics, it is important to consider aspects such as response time, analytical phase, and cost. Considering that it initially requires more training, as not everyone can manipulate the panels or acquisitions, it involves higher equipment costs, increased reagent use, greater complexity in analysis, and generally elevated costs. It is fair to consider how valid and necessary the incorporation of spectral flow cytometry into clinical diagnosis may be. Although we have not conducted a study of the economic impact, the panel for spectral flow cytometry entails a lower expenditure on antibodies since they are used only once, compared to the antibodies that form the backbone in conventional cytometry, which are used in four doses, one for each tube. Regarding time, titration and fine-tuning are the lengthiest but also necessary steps in order to maximize performance accuracy. Once this phase was optimized, we obtained similar response-times in our practice.

Extensive testing, reagent verification, and re-validation of cytometry assays are much needed in order to incorporate spectral flow cytometry into the clinical laboratory routinary workflow. Operational costs, ease of use, and the greater capacity of spectral cytometers will be important practical factors. Strategies for data storage and high dimension/automatization of data analysis will also be major bottlenecks to solve in the future before spectral flow cytometry becomes state-of-the-art for clinical diagnosis.

## Figures and Tables

**Table 1:** List of antibodies, fluorochromes, and final volumes for each flow cytometry platforms.

	Conventional Cytometry			Spectral Cytometry		
**Marker**	Clone	Fluorescence	Volume (μL)	Clone	Fluorescence	Volume (μL)
**CD2**	RPA-2.10	FITC	10	RPA-2.10	cF R685	2.5
**CD9**	M-L13	V450	5	HI9A	cF V450	2.5
**CD10**	HI10a	APC	5	HI10a	cF R780	2.5
**CD13**	WM15	PerCP-Cy5.5	5	WM15	cF R659	5
**CD15**	HI98	FITC	10	HI98	cF B548	5
**CD19**	SJ25C1	PE-Cy-7	5	SJ25C1	cF BYG781	2.5
**CD20**	L27	V450	5	2H7	cF B515	2.5
**CD22**	HIB22	APC	5	HIB22	cF BYG710	5
**CD24**	ML5	APC-Cy-7	5	SN3	cF BYG667	5
**CD33**	P67.6	APC	5	WM53	cF BYG610	5
**CD34**	8G12	PerCP-Cy5	5	4H11	cF BYG575	5
**CD38**	HB7	APC-Cy-7	5	HB7	cF B690	5
**CD45**	2B1	V500	5	HI30	cF R840	2.5
**CD52**	4C8	Alexa fluor 647	5	HIB168	cF V420	5
**CD58**	1C3	FITC	10	MEM-63	cF R720	5
**CD66c**	B6.2	PE	10	B6.2	BV786	2.5
				5E10C7	cF V780	2.5
**CD73**				AD2	cF B532	5
**CD81**	JS-81	FITC	10	M38	cF BYG750	5
**CD99**	TÜ12	PE	10	3B2/TA8	cF V610	5
**CD117**				104D2	BV711	5
**CD123**	7G3	APC	5	6H6	BV650	5
**CD304**				U21-1283	BV750	5
**HLA-DR**	L243	V450	5	L243	cF V505	5
**NG2**	9.2.27	PE	10	9.2.27	AF647	1.25

**Table 2:** Comparison of MFI of markers between single- and multi-tube staining.

Marker	Fluorochrome	Channel	MFI Median (SST)	SD (SST)	MFI Median (MST)	SD (MST)	*p*-Value
CD52	V420	V2	30,139.00	9644.26	26,676.00	7930.90	>0.99
CD9	V450	V3	183,603,66	186,295.87	156,581.33	164,926.01	0.25
HLA-DR	V505	V5	108,000.66	27,617.46	96,499.33	24,772.66	0.75
CD99	V610	V10	28,480.33	6392.92	22,126.00	6081.56	0.25
CD34	BYG575	B4	103,455.66	21,793.77	81,793.66	42,571.66	0.25
CD24	BYG667	B8	46,617.33	26,516.42	34,677.00	19,679.12	0.25
CD38	B690	B9	15,889.66	3390.38	12,068.00	2044.38	0.25
CD81	BYG750	B12	49,262.33	27,133.41	57,209.33	45,986.17	0.75
CD19	BYG781	B13	57,777.66	30,775.09	50,495.00	27,387.24	0.25
NG2	AF647	R2	15,126.50	727.61	21,702.00	527.50	0.5
CD10	R780	R7	132,714.00	144,348.86	141,025.00	115,726.26	>0.99
CD45	R840	R8	39,661.33	10,469.73	20,534.33	1632.07	0.25

**Table 3:** Paired comparison of each antibody in 27 primary samples. SP: spectral cytometry. CV: conventional cytometry.

	% of Discrepancy	SP > CV	SP < CV	Rate Overestimation	Rate Underestimation
**CD52**	0.13	1	1	0.06	0.06
**CD9**	0.00	0	0	0.00	0.00
**HLA-DR**	0.08	0	2	0.00	0.08
**CD99**	0.05	1	0	0.05	0.00
**CD66C**	0.04	0	1	0.00	0.04
**CD123**	0.13	0	3	0.00	0.13
**CD20**	0.11	2	1	0.07	0.04
**CD15**	0.04	1	0	0.04	0.00
**CD34**	0.07	1	1	0.04	0.04
**CD33**	0.04	0	1	0.00	0.04
**CD24**	0.11	3	0	0.11	0.00
**CD38**	0.00	0	0	0.00	0.00
**CD22**	0.17	2	2	0.08	0.08
**CD81**	0.00	0	0	0.00	0.00
**CD19**	0.00	0	0	0.00	0.00
**CD13**	0.15	1	3	0.04	0.12
**NG2**	0.00	0	0	0.00	0.00
**CD2**	0.00	0	0	0.00	0.00
**CD58**	0.15	3	1	0.11	0.04
**CD10**	0.11	3	0	0.11	0.00
**CD45**	0.46	12	0	0.46	0.00

## Data Availability

Data is available upon request.

## Supplementary Materials

The following supporting information can be downloaded at: https://www.mdpi.com/article/10.3390/cells13221891/s1, Supplementary Figure S1. Example of the analysis of a healthy marrow sample; Supplementary Figure S2. Spreading errors during panel optimization; Supplementary Figure S3. Strategy for to the cleaning/elimination of debris and doublets; Supplementary Table S1. List of markers commonly studied in B-cell acute lymphoblastic leukemia. Supplementary Table S2. Paired comparison between Spectral and Conventional flow cytometry.
