# Supplementary material for: Validation of a Spectral Flow Cytometry Single-Tube Panel for the Clinical Diagnosis and Follow-Up of Children and Adolescents with B-Cell Acute Lymphoblastic Leukemia
Source: Cells. 2024 Nov 15;13(22):1891. doi: 10.3390/cells13221891 (PMC11592797; doi:10.3390/cells13221891)
Supplement: Supplementary file 1 [file cells-13-01891-s001.zip › cells-3235094-supplementary.pdf]

Supplementary Information

Supplementary Figure 1.

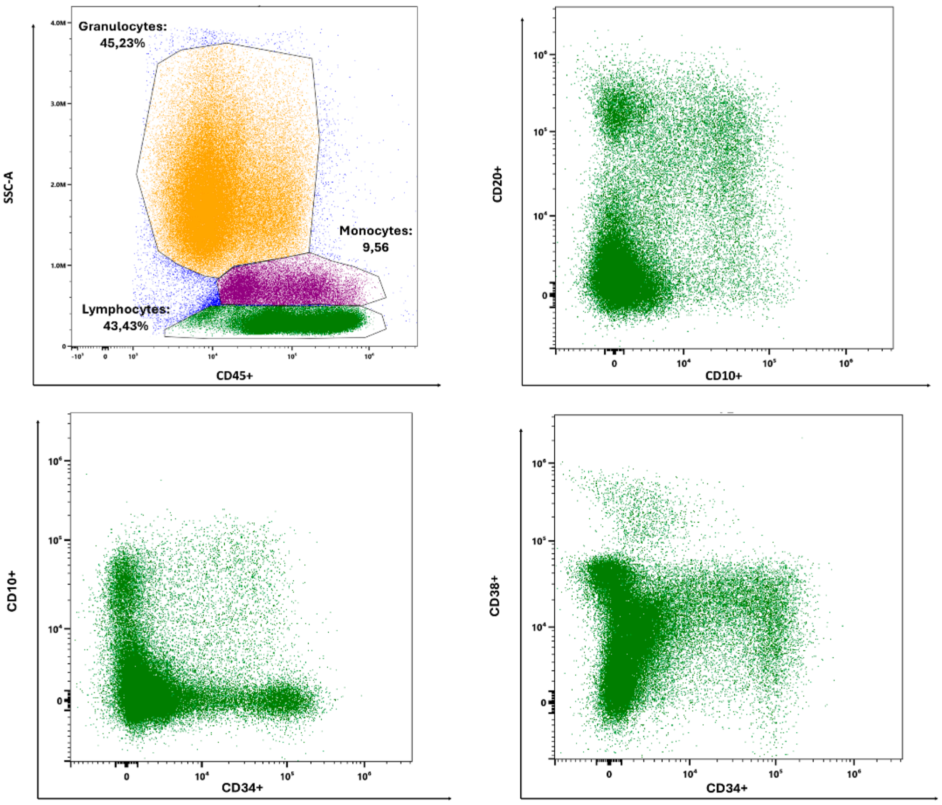

SP1. Example of the analysis of a healthy marrow sample. Major cell populations (up left) and B-cell maturation profiles in combination of classical markers.

Supplementary Figure 2.

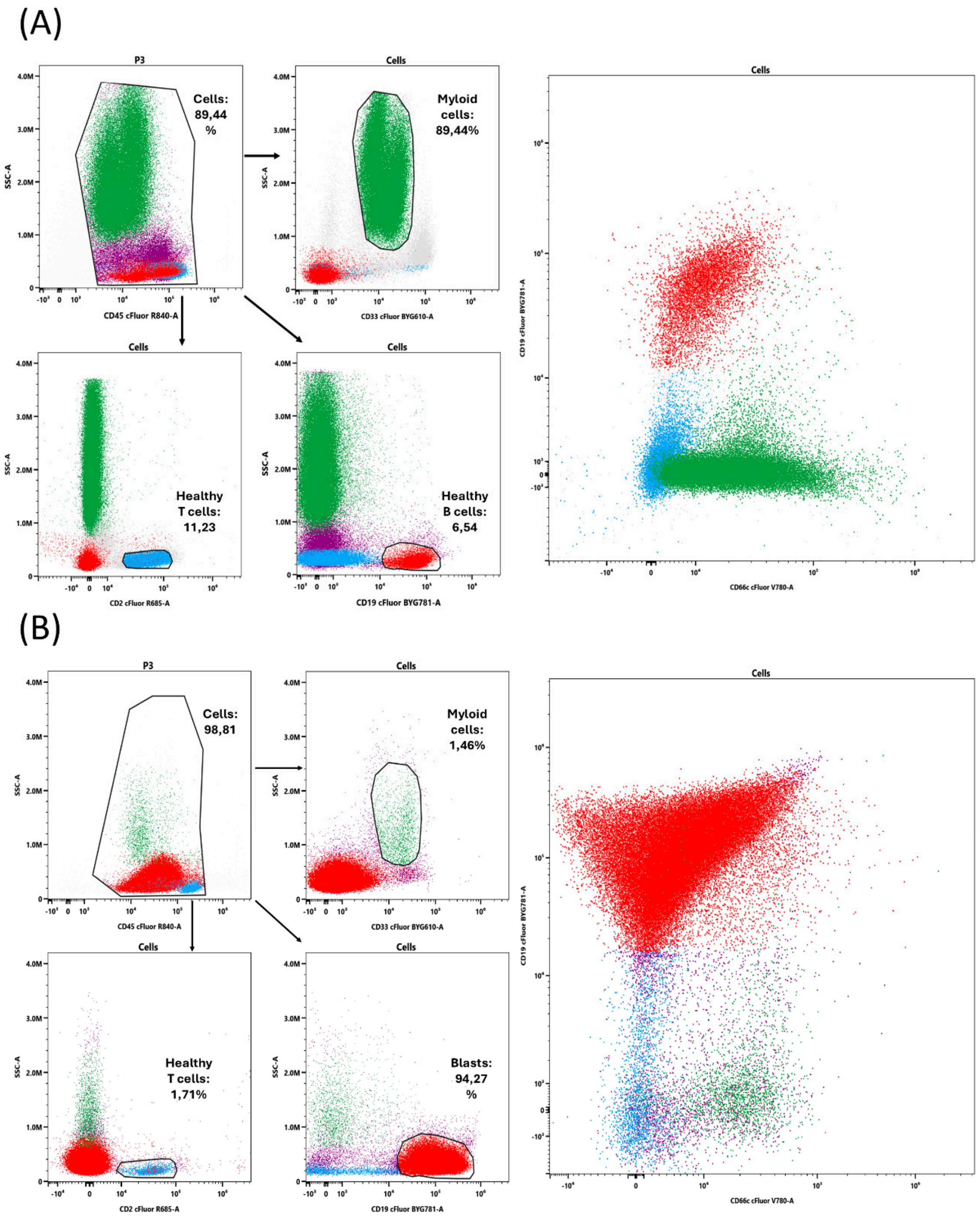

SP2. Spreading errors during panel optimization. (A) Healthy bone marrow and (B) B-ALL diagnostic samples, stained with B-ALL panel. T cells (Blue), Myeloid cells (green) and Healthy B cells (Red). Notice the spreading of the fluorescence related to the CD66c cFV780 antibody.

Supplementary Figure 3.

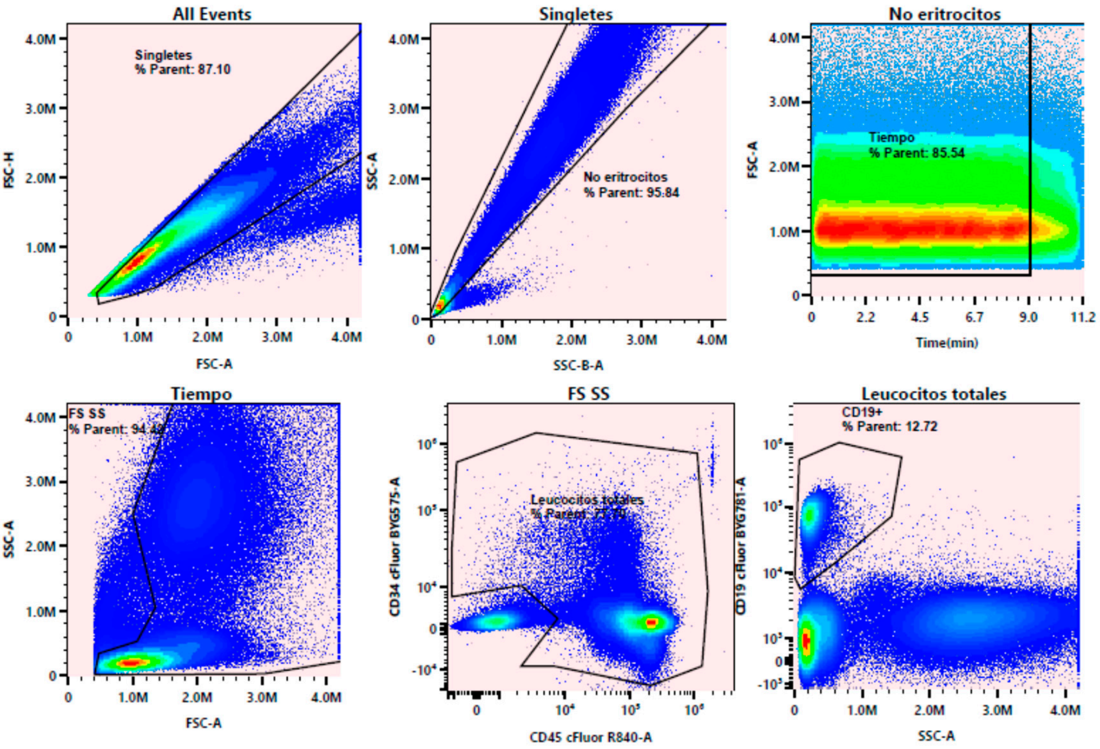

SP3. Strategy for to the cleaning/elimination of debris and doublets**.**

Supplementary Table 1.

RELATED TO	MARKER	ROLE
LINEAGE	MPO	CRITICAL FOR MPAL
	cyt-CD3	
	cyt-CD22	
	cyt-CD79a	
	cyt-IgM	
	Tdt	
NON-LINEAGE	CD-10	
	CD34	
	CD38	TARGETEABLE
	CD45	
	CD117	B-ALL / EPT DIAGNOSIS / MRD
	HLA-DR	
B-LINEAGE	CD19	
	CD20	
	CD22	MRD IN CASE OF BLINA / CART THERAPY
	CD24	
	CD81	
	KAPPA	B-ALL VS MATURE B
	LAMBDA	B-ALL VS MATURE B
ABERRANTS	CD73	MRD
	CD66c	MRD
	CD123	MRD
	CD304	MRD
	NG2	PRO-B / KMT2A REARRANGEMENTS
T-LINEAGE	CD2	
MYELOID	CD13	MRD
	CD15	PRO-B / MRD
	CD33	MRD

Supplementary Table 1. List of markers commonly studied in B-cell acute lymphoblastic leukemia. Cyt: intracellular. Tdt: Terminal deoxynucleotidyl transferase. NG2: Neural/glial antigen 2. MPAL: Mixed phenotype acute leukemia. EPT: early pre-T. MRD: measurable residual disease.

Supplementary Table 2.

		# 1	# 2	# 3	# 4	# 5	# 6	# 7	# 8	# 9	# 10	# 11	# 12	# 13	# 14	# 15	# 16	# 17	# 18	# 19	# 20	# 21	# 22	# 23	# 24	# 25	# 26	# 27
CD52	Spectral	2	2	1	2	1	3	2	3	3	3	2	2	2	3	2	2	1	2	2	4		3	4	1	2	1	1
	Canto	3		1	2	1	2	2	3		3	2	2	2	3	2	2	1	2									
CD9	Spectral	1	3	3	3	3	3	3	3	3	3	3	4	3	4	3	3	3	2	4	4	4	3	4	3	4	3	3
	Canto	1	3	3	3	3	3	3	3	3	3	3	4	3	4	3	3	3	2	4	4	4	3	4	3	4	3	3
HLA-DR	Spectral	3	3	3	3	3	3	3	3	4	3	2	1	3	3	3	3	3	3	3	3	4	4	4	3	2		3
	Canto	3	3	3	3	3	3	3	3		3	3	1	3	3	3	3	3	3		3	4	4	4	3	3	3	3
CD99	Spectral	4	3	3	3	3	3	3	3	3	1	3	3	3	3	3	3	3	1	3	3							
	Canto	4		3	3	3	3	3	3	2	1	3	3	3	3	3	3	3	1	3	3	3	3	3	3	3	3	3
CD66c	Spectral	1	3	3	1	1	1	1	1	1	1		3	3	3	1	1	1	3	3	4	3	1	4	1	3	3	1
	Canto	1	3	3	1	1	1	1	1		1		3	3	3	3	1	1	3	3	4	3	1	4	1	3	3	1
CD123	Spectral	1	1	2	3	1	3	3	1	2	2	1	3	3	4	1	2	3	3	3	4	3	3	2	1	3	3	
	Canto	1		2	3	1	3	3	2		3	1	3	3	4	2	2	3	3	3	4	3	3	2	1	3	3	3
CD20	Spectral	1	3	1	1	1	1	1	1	1	3	3	3	3	3	3	1	1	3	2	3	1	1	4	1	1	1	1
	Canto	1	3	1	1	1	1	1	1	2	3	3	3	3	3	3	1	1	1	2	2	1	1	4	1	1	1	1
CD15	Spectral	1	1	2	1	1	1	1	1	1	1	1	1	1	1	1	1	1	1	1	1	1	1	1	3	1	1	1
	Canto		1	1	1	1	1	1	1		1	1	1	1	1	1	1	1	1	1	1	1	1	1	3	1	1	1
CD34	Spectral	2	3	1	3	3	3	3	3	3	1	1	3	3	3	1	3	3	3	3	3	3	3	3	1	3	3	3
	Canto	3	3	1	3	3	3	3	3	3	1	1	3	3	3	1	3	3	3	2	3	3	3	3	1	3	3	3
CD33	Spectral	3	1	2	1	3	1	1	1	3	1	1	3	1	1	1	1	3	1	1	1	1	1	2	1	1		1
	Canto	3	1	3	1	3		1	1		1	1	3	1	1	1	1	3	1	1	1	1	1	2	1	1	1	1
CD24	Spectral	4	4	3	3	1	3	3	3	3	3	3	3	3	3	3	3	1	3	3	3	1	1	3	1	1	3	3
	Canto	4	4	3	3	1	3	3	3	3	3	3	3	1	3	3	3	1	2	3	3	1	1	2	1	1	3	3
CD38	Spectral	3	4	3	3	3	3	1	3	3	3	3	1	1	3	3	3	3	3	3	3	3	3	3	3	3	3	3
	Canto	3	4	3	3	3	3	1	3	3	3	3	1	1	3	3	3	3	3	3	3	3	3	3	3	3	3	3
CD22	Spectral	3	2	3	2	2	2	3	2	2	3	2	2	2	2	2	2	1	3	2	3	2	2			1	2	3
	Canto	2	2	3	2	2	2	1	3		3	2	2	2	2	2	2	1	2	3	3	2	2	3	3	1	2	3
CD81	Spectral	3	3	3	3	3	3	3	3	3	3	3	3	3	3	3	3	3	3	3	3						3	3
	Canto	3		3	3	3	3	3	3		3	3	3	3	3	3	3	3	3	3	3	3	3	3	3	3	3	3
CD19	Spectral	3	3	3	3	3	3	3	3	3	3	3	3	3	3	3	3	3	3	3	3	3	3	3	3	3	3	3
	Canto	3	3	3	3	3	3	3	3	3	3	3	3	3	3	3	3	3	3	3	3	3	3	3	3	3	3	3
CD13	Spectral	1	1	1	2	2		1	1	3	1	1	1	1	1	1	1	1	1	1	1	1	1	1	1	1	1	1
	Canto	1	1	1	1	2		1	1		2	1	1	1	1	1	1	1	2	3	1	1	1	1	1	1	1	1
NG2	Spectral	1	1	1	1	1	1	1	3	1	1	1	1	1	1	1	1	3	1	1	1	1	1	1	3	1	1	1
	Canto	1	1	1	1	1	1	1	3		1	1	1	1	1	1	1	3	1	1	1	1	1	1	3	1	1	1
CD2	Spectral	1	1	1	1	1	1	1	1	1	3	1	1	1	1	1	1	1	3	1	1	1	1	1	1	1	1	1
	Canto	1		1	1	1	1	1	1		3	1	1	1	1	1	1	1	3	1	1	1	1	1	1	1	1	1
CD58	Spectral	3	1	1	3	3	1	3	1	1	1	3	3	1	3	3	3	3	3	3	3	3	3	3	1	3	1	1
	Canto	1	1	1	3	3	1	3	1	3	1	3	3	1	1	3	1	3	3	3	3	3	3	3	1	3	1	1
CD10	Spectral	4	4	1	3	3	4	3	1	3	3	3	3	4	4	3	4	1	3	4	3	3	4	4	3	4	4	3
	Canto	4	4	1	3	3	4	3	1	3	3	3	3	3	4	3	4	1	3	3	3	3	4	4	1	4	4	3
CD45	Spectral	3	3	3	3	2	2	2	3	2	3	2	2	2	2	3	2	2	2	2	1	3	2	2	2	3	1	1
	Canto	3	2	3	1	2	1	1	3	2	2	1	2	1	1	2	1	2	2		1	2	2	2	2	2	1	1

Supplementary Table 2. Paired comparison between Spectral and Conventional flow cytometry. Expression levels for each marker were catalogued as 1 (negative), 2 (dimly positive), 3 (positive), and 4 (highly positive).
